# Validation of the Japanese version of the EORTC hepatocellular carcinoma-specific quality of life questionnaire module (QLQ-HCC18)

**DOI:** 10.1186/1477-7525-10-58

**Published:** 2012-05-31

**Authors:** Naoko Mikoshiba, Ryosuke Tateishi, Makoto Tanaka, Tomoko Sakai, Jane M Blazeby, Norihiro Kokudo, Kazuhiko Koike, Keiko Kazuma

**Affiliations:** 1 Department of Adult Nursing, Division of Health Sciences and Nursing, Graduate School of Medicine, The University of Tokyo, 7-3-1 Hongo, Bunkyo-ku, Tokyo, 113-0033, Japan; 2 Department of Gastroenterology, The University of Tokyo, 7-3-1 Hongo, Bunkyo-ku, Tokyo, 113-0033, Japan; 3 Surgical research unit, School of Social and Community Medicine, University of Bristol, Canynge Hall, 39 Whatley Road, Bristol, BS8 2PS, UK; 4 Department of Surgery, Hepato-Bilialy-Pancreatic Surgery Division, Artificial Organ and Transplantation Division, The University of Tokyo, 7-3-1 Hongo, Bunkyo-ku, Tokyo, 113-0033, Japan

**Keywords:** EORTC QLQ-HCC18, FACT-Hep, Hepatocellular carcinoma, Quality of life, Questionnaire

## Abstract

**Background:**

This study examined the measurement properties of the Japanese version of the European Organisation for Research and Treatment of Cancer (EORTC) Hepatocellular Carcinoma-Specific Quality of Life Questionnaire (QLQ-HCC18).

**Methods:**

EORTC quality of life (QOL) translation guidelines were followed to create a Japanese version of the EORTC QLQ-HCC18. This was then administered to 192 patients with hepatocellular carcinoma along with the EORTC QLQ-C30 and FACT-Hep questionnaires. Tests for reliability and validity were conducted including comparison of scores between the EORTC and FACT questionnaire and detailed assessment of the new scales and items in clinically distinct groups of patients.

**Results:**

Multi-trait scaling analysis confirmed three putative scales in the QLQ-HCC18, fatigue, fever and nutrition. Cronbach’s alpha for these scales were between 0.68 and 0.78. The QLQ-HCC18 scales correlated with scales measuring similar items in the FACT-Hep and the questionnaire was stable over time with an intra-class correlation score of 0.70 for almost all scales. The questionnaire had the ability to distinguish between patients with different Karnofsky Performance Status, and Child-Pugh liver function class.

**Conclusions:**

The Japanese version of EORTC QLQ-HCC18 is a reliable supplementary measure to use with EORTC QLQ-C30 to measure QOL in Japanese patients with hepatocellular carcinoma.

## Background

Hepatocellular carcinoma (HCC) is the most common malignancy in the world, accounting for more than half a million new cases annually [1,2]. The highest incidence rates are in eastern and south-eastern Asia, western and central Africa [2]. The incidence is low in most developed countries, however, Japan has a very high prevalence of HCC, and 70% are caused by hepatitis C viruses [3]. Although the 5-year survival rates of up to 60 to 70% can be achieved in well-selected patients, the recurrence rate remains very high [4,5]. The 5-year recurrence rate after potentially curative liver resection is up to 80% [4-6]. In countries such as Japan, where cadaveric donor organs are scarce, application of liver transplantation is limited [7,8]. Thus, most patients with HCC undergo repeated non-transplant treatments such as surgical resection, percutaneous radiofrequency ablation and embolization. Although survival data and information about the side effects of treatment are widely available, much less is known about how treatment for HCC impacts upon the patients’ quality of life (QOL). Given the time course of the disease, and the burden of repeated treatment, there are increasing concerns about QOL associated with HCC. When deciding upon treatment, consideration of QOL outcomes could be as important as survival. However, there are no HCC-specific QOL questionnaires in Japan.

At present, there are two disease-specific QOL questionnaires for evaluating the QOL of patients with HCC. One is the European Organization for Research and Treatment of Cancer (EORTC) Quality of Life Group questionnaire, the QLQ-HCC18, and the other is the Functional Assessment of Cancer Therapy (FACT) Hepatobiliary (FACT-Hep) questionnaire [9,10]. As they are disease-specific, they are combined with generic questionnaires such as the QLQ-C30 and FACT generic questionnaires, respectively, to produce a generic and a specific QOL assessment [11,12]. The major difference between FACT-Hep and EORTC QLQ-HCC18 is that FACT-Hep targets not only patients with HCC but also patients with pancreatic, biliary and metastatic liver cancer, whereas the QLQ-HCC18 is designed specifically for patients with HCC. Currently there is a lack of published data demonstrating the measurement properties of EORTC QLQ-HCC18.

The objective of this study, therefore, was to develop a Japanese version of EORTC QLQ-HCC18, and to validate its measurement properties in patients with HCC.

## Methods

### Translation of the Japanese version of EORTC QLQ-HCC18

The EORTC guidelines for translation of the QLQ-HCC18 was followed and authorized by the EORTC [13]. This included a forward/backward translation of EORTC QLQ-HCC18. The original English version was translated into Japanese by two independent translators who were native Japanese speakers with proficiency in English. The research coordinator compared the two forward translations and checked them for any discrepancies. The discrepancies between the two translations were discussed with the translators until we agreed on one provisional forward translation. This forward translation was then back translated into English by two independent translators who were native speakers of English with proficiency in Japanese. The English back translations and the original English version were compared to assure that there were no differences in the meaning of the questions in the questionnaires. The provisional Japanese version was pilot tested on 10 patients diagnosed with HCC who had satisfied the following eligibility criteria: (1) age > 20 years; (2) ability to communicate in Japanese; (3) ability to participate in this study, as judged by an attending doctor; (4) confirmation of medical diagnosis; (5) no other concurrent malignancy; and (6) consent to participate in this study. The pilot test was conducted according to the manual provided by EORTC [13] as of June 2008. The average time necessary for completing the QLQ-HCC18 was less than 5 minutes and the questionnaire was well understandable and acceptable in most patients. Results of the translation and the pilot study were reviewed by the EORTC translation coordinator and the original author of QLQ-HCC18, to ensure the content and applicability was maintained, and the EORTC QLQ-HCC18 Japanese version was authorized by the EORTC Quality of Life Group. The Japanese version of EORTC QLQ-HCC18 was used in this validation study.

### Data collection

This study recruited 200 patients diagnosed with HCC at The University of Tokyo Hospital, one of the largest referral centers for treatment of HCC in Japan, and written consent was obtained. Patients were recruited between July 2008 and November 2008. The eligibility criteria were the same as for pilot testing. Patients completed each of the three questionnaires: EORTC QLQ-C30, QLQ-HCC18, and FACT-Hep, and a questionnaire about demographic characteristics. To confirm test-retest reliability of the Japanese version of QLQ-HCC18, patients with stable disease were invited to complete QLQ-HCC18 for a second time after two weeks. Medical data were collected by review of medical care records. The researcher checked for absent responses after receiving the questionnaire and wherever possible asked the patients to respond to the missing items. This study was conducted with the approval of the ethics committee of The University of Tokyo.

### Measurements

The EORTC QLQ-C30 core questionnaire (version 3.0) is a generic QOL measure for cancer patients, and comprises a global health status/QOL scale, five multi-item functional scales, three multi-item symptom scales and single items for the assessment of symptoms and the financial impact of disease and treatment [11]. The reliability and validity of the Japanese version of the EORTC QLQ-C30 has been demonstrated [14].

EORTC QLQ-HCC18 is an 18-item HCC-specific supplemental module developed to augment QLQ-C30 and to enhance the sensitivity and specificity of HCC-related QOL issues [9]. EORTC QLQ-HCC18 was developed in four stages on the basis of the EORTC guidelines for scale development [9]. Briefly, items were created during phase one after conducting a literature review and interviewing 32 patients with HCC from four different countries as well as 10 health professionals. In phase two, a preliminary questionnaire was constructed using the EORTC item bank as a reference. In phase three, a pretest was administered to 158 patients with HCC from three countries to examine receptivity and relevance. The original questionnaire is from the end of phase three. The hypothesized scale structure and single items address aspects of chronic liver disease (nutrition, jaundice, fever, abdominal swelling), as well as QOL issues specific to the primary tumor and its treatment (fatigue, body image, pain).

The original English version contains six multi-item scales addressing fatigue, body image, jaundice, nutrition, pain and fever, as well as two single items addressing sexual life and abdominal swelling. The scales and items are linearly transformed to a 0 to 100 score, where 100 represents the worst status. An international field test (the phase 4 part of questionnaire development) is currently being conducted to examine the validity and reliability of the scores in several countries.

The reliability and validity of the original version of FACT-Hep, another hepatobiliary cancer-specific scale, has been demonstrated [10]. FACT-Hep is a 45-item self-report instrument that comprises 27 FACT General (FACT-G) items and an 18-item hepatobiliary subscale. The Japanese version of the 18-item hepatobiliary subscale was used in this study as a comparison instrument. All items are scored from 0 to 4, with higher scores indicating better QOL.

### Data analysis

Multi-trait scaling analyses [15] evaluated the scale structures of QLQ-HCC18. This technique is used to test for item convergent and discriminant validity, and is based on the examination of item-scale correlations. The Pearson correlations of an item with its own scale (corrected for overlap) and other scales were calculated. Evidence of item convergent validity was defined as a correlation above 0.40 with its own scale. Evidence of item discriminant validity was based on a comparison of correlation of an item with its own scale and with other scales. Scaling success for any scale is defined as the number of convergent correlation coefficients significantly higher than the discriminant correlation coefficient divided by the total number of correlations. The mean scale and item scores were also calculated, and a frequency analysis was performed.

The following psychometric aspects were assessed: reliability, i.e., internal consistency and test-retest reliability; validity: known group comparison, and correlation analyses with the FACT-Hep.

The internal consistency reliability of the multi-item questionnaire scales was assessed by Cronbach’s alpha coefficient. Preferable reliability was indicated by coefficient greater than 0.70. The test-retest reliability of the scales and single items was assessed by the intra-class correlation coefficient. Scale discriminant validity (clinical validity) was tested by known group comparisons to assess whether the questionnaire scores were able to discriminate between subgroups of patients differing in clinical status by using the Student t-test. The Karnofsky Performance Status (KPS) and Child-Pugh grade for clinical parameters were employed to form mutually exclusive patient subgroups. Higher scores in KPS signify better performance status. Liver function becomes worse in alphabetical order of Child-Pugh grade A, B, C. We hypothesized that scores of QLQ-HCC18 are low in patients with better performance status (KPS 80–100) and better liver function (Child-Pugh class A). Convergent validity was tested first by multi-trait analyses, and we then conducted another convergent validity test by correlation analyses with FACT-Hep. Pearson’s correlation coefficient was used to examine the correlation between similar items in FACT-Hep and QLQ-HCC18. We hypothesized that if Pearson’s correlation coefficients were more than 0.40 between scales, they were conceptually related. *P* < 0.05 was considered as statistically significant. Statistical analyses were performed using SAS software (SAS for Windows, release 9.1; SAS Institute Inc., Cary NC, USA).

## Results

### Participants

Responses were obtained from 192 patients (eight non responders), and 139 completed the test-retest questionnaire two weeks after the first assessment.

Socio-demographic and clinical characteristics at baseline are shown in Table 1. Most patients were male (64.1%), had good performance status (86.5%) and had good liver function (66.2%).

**Table 1:** Socio-demographic and clinical characteristics of the study subjects (n = 192)

**Male gender**	**122 (63.5)**
Age, y*	68.1 (8.5)
Employed full time or part-time	77 (40.1)
Post compulsory education or above	155 (80.7)
Married or living with partner	151 (78.6)
Karnofsky Performance status	
80-100	166 (86.5)
Comorbid liver disease	
Hepatitis C virus	126 (65.6)
Hepatitis B virus	38 (19.8)
Other or unknown	28 (14.6)
Child-Pugh class	
A	127 (66.1)
B	53 (27.6)
C	12 (6.3)
Cancer stage	
Stage I / II	161 (83.9)
Stage III / IV	31 (16.1)
Time since diagnosis, month*	39.6 (34.5)
Past medical history^†^	
Hepatectomy	48 (25.0)
Percutaneous ablation	137 (71.4)
Chemoembolization	64 (33.3)
Systemic therapy	1 (0.5)
No medical history	13 (6.8)

Values are numbers (%) otherwise specified. * Data was expressed as mean (standard deviation). ^**†**^ Some patients underwent multiple treatments.

### Psychometric testing

We initially performed multi-trait scaling analyses for the putative scale structure, and the results showed that the original two-item scale of body image and jaundice had low convergent and discriminant validity. After discussion with the original author of QLQ-HCC18 (JMB) we decided to split the scale into single items. The tests were then performed on the remaining scales and four single items. Results of the multi-trait scaling analyses are shown in Table 2. A summary of the multi-trait scaling analysis and internal consistency is shown in Table 3. The convergent correlation coefficient of the scales for fatigue, nutrition and fever varied from 0.23 to 0.75, and the scaling success rate ranged from 87% to 100%. Cronbach’s alpha coefficient of these scales was satisfactory, ranging from 0.68 to 0.78. The convergent correlation coefficient of the scales for pain was 0.25, and the scaling success rate was 50%. Cronbach’s alpha coefficient of this scale was 0.37.

**Table 2:** Item-scale correlations for multi-trait scaling analyses of the EORTC QLQ-HCC18

		**Hypothesized scales of the EORTC QLQ-HCC18**^**†**^			
**Item**		**Fatigue**	**Nutrition**	**Pain**	**Fever**
**Fatigue**					
Item46	Have you been less active than you would like to be?	0.68^†^	0.57	0.29	0.32
Item45	Have you found it difficult to keep going or to finish things you started?	0.75^†^	0.55	0.32	0.35
Item47	Have you needed to sleep during the day?	0.44^†^	0.29	0.24	0.14
**Nutrition**					
Item31	Did you feel thirsty?	0.42	0.50^†^	0.30	0.23
Item32	Have you had problems with your sense of taste?	0.30	0.50^†^	0.18	0.18
Item42	Have you worried about getting enough nourishment?	0.45	0.54^†^	0.31	0.51
Item43	Have you felt full up too quickly after beginning to eat?	0.48	0.44^†^	0.30	0.27
Item44	Have you worried about your weight being too low?	0.21	0.23^†^	0.04	0.28
**Pain**					
Item38	Have you had pain in your shoulder?	0.20	0.15	0.25^†^	0.11
Item39	Have you had abdominal pain?	0.39	0.48	0.25^†^	0.36
**Fever**					
Item40	Have you had fevers?	0.34	0.46	0.27	0.52^†^
Item41	Have you had chills?	0.23	0.31	0.19	0.52^†^

Correlations marked † were corrected for overlap.

**Table 3:** Convergent and discriminant validity and internal consistency reliability for the EORTC QLQ-HCC18

**Scale**	**No. of items per scale**	**Convergent validity (range of correlations)**	**Discriminative validity (range of correlations)**	**Scaling success**^**†**^	**Scaling success rate**^**‡**^	**Internal consistency reliability (Cronbach’s α)**
Fatigue	3	0.44-0.75	0.14-0.57	9/9	100	0.78
Nutrition	5	0.23-0.54	0.04-0.51	13/15	87	0.68
Pain	2	0.25	0.11-0.48	3/6	50	0.37
Fever	2	0.52	0.15-0.46	6/6	100	0.68

^†^ Number of convergent correlations significantly higher than discriminant correlations divided by total number of correlations.

^‡^ Scaling success rate is the previous column as a percentage.

The results of the descriptive statistics of the putative scales/single items and test-retest reliability on the questionnaire are shown in Table 4. The intra-class correlation coefficients of the scales varied between 0.67 and 0.88. Ninety-four percent of the patients answered ‘not at all’ to item 36, which asked patients whether they were concerned by their skin or eyes being yellow. Responses to item 48, which asked about sexual function, were missing in seven patients (3.6%).

**Table 4:** Descriptive statistics of the EORTC QLQ-C30 and the QLQ-HCC18 and test-retest reliability

**Scale**	Score*	**Intraclass correlation coefficient**^**†**^
**The QLQ-HCC18**		
Scales^‡^		
Fatigue	25.6 ± 22.2	0.82
Nutrition	12.7 ± 14.1	0.88
Pain	13.5 ± 17.1	0.80
Fever	5.3 ± 12.9	0.67
Single items		
Body Image 1 (item33)	34.0 ± 31.9	0.73
Body Image 2 (item35)	21.1 ± 27.7	0.70
Jaundice 1 (item36)	2.77 ± 2.45	0.79
Jaundice 2 (item37)	22.2 ± 27.4	0.82
Abdominal Swelling	15.8 ± 23.6	0.78
Sexual Interest	12.6 ± 25.2^§^	0.77^‖^
The QLQ-C30		
Scales		
Physical^¶^	84.9 ± 16.9	-
Role^¶^	84.1 ± 22.3	-
Cognitive^¶^	84.2 ± 17.0	-
Emotional^¶^	79.6 ± 20.5	-
Social^¶^	86.1 ± 20.6	-
Global QOL^¶^	66.5 ± 21.63	-
Fatigue^‡^	30.9 ± 21.5	-
Nausea/Vomiting^‡^	1.74 ± 6.4	-
Pain^‡^	12.8 ± 20.6	-
Single items^‡^		
Dyspnea	15.8 ± 21.6	-
Sleep Disturbance	21.5 ± 27.5	-
Appetite Loss	12.7 ± 22.5	-
Constipation	14.2 ± 23.9	-
Diarrhea	7.81 ± 16.4	-
Financial Impact	14.6 ± 23.3	-

Data were expressed as mean ± standard deviation. * Score range 0 to 100. ^†^ Data were assessed in 130 patients. ^‡^Higher score indicates lower QOL. ^§^ Data were assessed in 185 patients. ^‖^ Data were assessed in 127 patients. ^¶^ Higher score indicates higher QOL.

Results of the known group comparisons are shown in Table 5. Patients with poorer performance status (KPS of 70 or lower) reported significantly higher (worse) scores for all scales except for abdominal swelling and sexual interest than those with better performance status (KPS of 80–100). Patients with worse liver disease (Child-Pugh classes B and C) reported significantly higher (worse) scores for all scales except for body image and pain than those with better liver function (Child-Pugh class A).

**Table 5:** Known group comparison of differences in mean scores of scales and items

	**Karnofsky Performance status score**			**Child-Pugh grade**		
	**100-80**	**<80**	**t-test**	**A**	**B and C**	**t-test p value**
	**n = 166**	**n = 26**	**p value**	**n = 127**	**n = 65**	
Scales^**†**^						
Fatigue	21.0 ± 17.8	54.7 ± 25.9	< 0.001	22.0 ± 20.4	32.7 ± 24.1	0.001
Nutrition	10.8 ± 11.4	24.9 ± 22.0	< 0.001	10.9 ± 12.0	16.2 ± 17.1	0.02
Pain	11.6 ± 15.7	25.6 ± 20.7	0.003	12.3 ± 17.2	15.9 ± 16.8	0.17
Fever	4.3 ± 10.2	11.5 ± 23.0	0.003	3.5 ± 9.1	8.7 ± 17.7	0.02
Single items^**†**^						
Body Image 1(item33)	30.7 ± 29.6	55.1 ± 38.8	0.004	29.7 ± 30.3	42.6 ± 33.6	0.01
Body Image 2 (item35)	19.5 ± 26.5	32.1 ± 33.3	0.03	19.7 ± 25.3	24.1 ± 32.0	0.34
Jaundice 1 (item36)	1.2 ± 6.2	12.8 ± 28.4	0.04	0.8 ± 5.1	6.7 ± 19.7	0.02
Jaundice 2 (item37)	17.7 ± 23.4	51.3 ± 33.0	< 0.001	16.5 ± 23.7	33.3 ± 30.6	0.002
Abdominal swelling	14.1 ± 21.2	27.0 ± 34.0	0.07	13.1 ± 21.5	21.0 ± 26.7	0.04
Sexual interest	12.0 ± 24.1^‡^	16.7 ± 31.6	0.47	9.3 ± 21.1^§^	19.0 ± 30.9^‖^	0.03

Data were expressed as mean ± standard deviation unless otherwise specified. * p < 0.05, ^**†**^Higher score indicates worse QOL

^‡^ missing in 7, ^§^ missing in 5, ^‖^ missing in 2.

Results of convergent validity are shown in Table 6. The QLQ-HCC18 Japanese version scales had an acceptable correlation (coefficient value over 0.40) with similar items in FACT-Hep except for items of weight loss, appetite and activity.

**Table 6:** Pearson’s correlation coefficients between scales in the QLQ-HCC18 and the FACT-Hep

	**The FACT-Hep**												
**The QLQ-HCC18**	**Abdominal Swelling**	**Weight Loss**	**Appetite**^****†****^	**Appearance**	**Fatigue**	**Activity**^**†**^	**Jaundice**	**Fever**	**Itching**	**Taste**	**Chill**	**Thirsty**	**Abdominal Pain**
Fatigue	0.33	0.26	0.2	0.42*	0.59*	**0.26**	0.26	0.3	0.43*	0.32	0.17	0.37	0.42*
Body Image 1 (item33)	0.31	**0.32**	−1.7	0.46*	−0.08	0.17	0.3	0.3	0.25	0.25	0.23	0.45	0.38
Body Image 2 (item35)	**0.39**	0.01	−0.05	0.4*	0.1	0.07	0.18	0.31	0.2	0.3	0.12	0.23	0.47
Jaundice 1 (item36)	0.25	0.19	−0.07	0.2	−0.13	0.73	0.53*	0.3	0.12	0.18	0.56	0.28	0.3
Jaundice 2 (item37)	0.25	0.05	−0.05	0.31	0.45	−0.01	0.18	0.28	0.83*	0.33	0.26	0.35	0.37
Nutrition	0.32	**0.34**	**0.31**	0.32	0.38	0.23	0.31	0.28	0.26	0.44*	0.23	0.68*	0.33
Pain	**0.32**	0.11	0.14	0.28	0.32	0.14	0.21	0.26	0.34	0.15	0.2	0.3	0.40*
Fever	0.25	0.26	0.13	0.18	0.25	0.09	0.29	0.72*	0.35	0.18	0.61*	0.22	0.27
Abdominal Swelling	0.43*	0.07	0.03	0.16	0.29	0.06	0.23	0.3	0.3	0.15	0.28	0.24	0.42*
Sexual Interest^‡^	0.26	0.14	−0.06	0.25	0.24	0.08	0.06	0.12	0.12	0.06	0.12	0.13	0.24

* indicates Pearson’s correlation coefficient larger than 0.4, **_**Underline indicates a pair of scales that should correlate theoretically, ^**†**^ Reverse scoring, ^‡^ Data were assessed in 185 patients.

## Discussion

This study describes psychometric testing of the Japanese version of the QLQ-HCC18 questionnaire, which is an HCC-specific module of EORTC QLQ-C30. The overall results show that this questionnaire is reliable and has acceptable measurement properties for use with the QLQ-C30 to assess health-related QOL in Japanese patients with HCC.

Assessment of QOL in cancer patients is optimally performed with a combination of a generic questionnaire and a disease-specific questionnaire to ensure that common problems are uniformly detected and reported as well as specific issues related to disease site and treatment. This framework for QOL assessment has been adopted and popularized by the EORTC Quality of Life Group and the Functional Assessment of Chronic Illness Therapy (FACIT) Organization. For patients with primary and secondary liver tumors, cholangiocarcinoma or pancreatic cancer, the FACIT system has developed a single hepatobiliary-pancreatic module [10]. The EORTC QOL Group has, however, focused in more depth on the specific clinical experiences within each disease site and therefore developed separate modules for pancreatic, primary and secondary liver cancer. The separate modules may be clinically more sensitive than a single questionnaire, although this has not yet been formally examined. A second advantage of the EORTC QLQ-HCC18 is that it provides subscale scores for different domains of functioning. FACT-Hep generates only a total score, which may obscure findings in particular problem areas. EORTC QLQ-HCC18 possesses a multi-dimensional QOL assessment that may be more useful for clinicians to direct therapy. A final advantage of the EORTC QLQ module is that it was specifically developed for use in international trials; a large database will soon be available to facilitate comparisons across studies, and there is some assurance of cross-cultural suitability.

In this study, we tested the reliability and validity, including internal consistency reliability, test-retest reliability, convergent and discriminant validity, known group comparison, of the Japanese version of QLQ-HCC18. In the descriptive statistics and frequency analyses, the item assessing problems related to jaundice showed low scores. This was because few patients were jaundiced at the time of the data collection. In addition, because the Japanese belong to a race with a yellowish skin complexion, jaundice tends to be masked. The results of multi-trait scaling analyses (convergent and discriminant validity), had a good scaling success rate and acceptable Cronbach’s alpha (internal consistency reliability) except for the scale for pain, which had a low scaling success rate and a low Cronbach’s alpha. One reason for this may be because shoulder and abdominal pain are not necessarily related symptoms that occur simultaneously. Furthermore, although pain scales have been created in anticipation of pain caused by cancer treatment and progression, few patients had advanced cancer. The nutrition scale had a high rate of success. However, the convergent validity of the item termed “concern about low weight” was below the standard value. The nutrition scale was assumed to involve problems caused by impaired liver function, but items regarding weight loss may also have been affected by cancer progression. Patients included in the original article and patients in this study had almost identical liver function, but the extent of cancer progression differed, and many of our patients had cancer that was detected at an earlier stage. The results of test-retest reliability showed good intra-class correlation coefficients for most scales. Results of known group comparisons showed that the module had the ability to assess differences between groups with different clinical characteristics in almost all of the scales, showing the module has clinical validity. We confirmed good correlations between the groups for most scales/single items in the two questionnaires (QLQ-HCC18 and FACT-Hep). However, correlations between items of weight loss, appetite, and activity in FACT-Hep and corresponding scales in QLQ-HCC18 were low. This may have occurred because of the reverse scoring used in appetite and activity items in FACT-Hep which may have led to confusion.

While the results show the Japanese version of EORTC QLQ-HCC18 is a reliable instrument, some caution is necessary. First, these results on the QLQ-HCC18 are preliminary as this study was performed in a single institution using the Japanese version, few patients with severe cirrhosis or advanced disease were recruited, and no patient had undergone liver transplantation, which may limit the generalizability of the findings.

Second, this study did not address longitudinal construct validity and responsiveness for clinical validity. In future work, the Japanese version of EORTC QLQ-HCC18 should be performed in multicenter facilities to confirm the generalizability of the findings and to increase the number of liver transplantation groups and more severely ill patients. Furthermore, testing the sensitivity of the instrument to changes over time is needed to evaluate treatment effects.

There are currently a variety of treatment options for patients with HCC. Molecular targeted therapy for HCC has recently been introduced [16], and this will lead to increased demand for evaluating the QOL in more detail. In addition, Japanese patients with HCC are older than in other countries, which make the Japanese version of QLQ-HCC18 particularly valuable because treatment effects on QOL are more important in older patients.

## Conclusion

This study showed that the Japanese version of the EORTC QLQ-HCC18 demonstrated evidence for the measurement properties of the questionnaire. These results suggest that it would be a reliable instrument for measuring QOL in patients with HCC in Japan.

## Abbreviations

QOL: Quality of Life; EORTC: European Organisation for Research and Treatment of Cancer; QLQ: Quality of life questionnaire; HCC: Hepatocellular carcinoma; KPS: The Karnofsky Performance Status; RFA: Radio frequency ablation.

## Competing interests

The authors declare that they have no competing interests.

## Authors’ contributions

NM, TS, MT, RT, JMB, NK and KK conceptualized the rationale and design of the study. NM, TS, MT, RT, JMB and KK conducted scale development. NM and TS presented this study to patients and collected data. NM, TS, MT, RT, JMB and KK conducted statistical analyses and interpreted the data. NM, RT, JMB drafted the manuscript. All authors read and approved the final manuscript.
